# The impact of apraxia and neglect on early rehabilitation outcome after stroke

**DOI:** 10.1186/s42466-022-00211-x

**Published:** 2022-09-26

**Authors:** S. Latarnik, J. Stahl, S. Vossel, C. Grefkes, G. R. Fink, P. H. Weiss

**Affiliations:** 1 Department of Neurology, Faculty of Medicine, University Hospital Cologne, University of Cologne, 50924 Cologne, Germany; 2 Cognitive Neuroscience, Institute of Neuroscience and Medicine (INM-3), Forschungszentrum Jülich, Jülich, Germany; 3 Department of Psychology, Faculty of Human Sciences, University of Cologne, Cologne, Germany

**Keywords:** Apraxia, Neglect, Stroke, Outcome, Rehabilitation

## Abstract

**Background:**

This study aims to characterize the impact of apraxia and visuospatial neglect on stroke patients’ cognitive and functional outcomes during early rehabilitation. Prior work implies an unfavorable effect of visuospatial neglect on rehabilitation; however, previous findings remain ambiguous and primarily considered long-term effects. Even less is known about the impact of apraxia on rehabilitation outcomes. Although clinicians agree on the significance of the first few weeks after stroke for the course of rehabilitation, studies exploring the impact of neglect and apraxia in this early rehabilitation period remain scarce.

**Methods:**

Based on a screening of 515 hospitalized stroke patients from an early rehabilitation ward, 150 stroke patients (75 left-hemispheric strokes, 75 right hemispheric strokes) fulfilled the inclusion criteria and were enrolled in this observational, longitudinal study. The patients’ cognitive and functional statuses were documented at admission to the early rehabilitation ward and discharge. Also, detailed apraxia and neglect assessments were performed at midterm. The predictive values of age and apraxia and neglect severity (as reflected in two components from a principal component analysis of the neglect and apraxia assessments) for cognitive and functional outcomes at discharge were evaluated by multiple regression analyses.

**Results:**

Besides the expected influence of the respective variables at admission, we observed a significant effect of apraxia severity on the cognitive outcome at discharge. Moreover, neglect severity predicted the Early Rehabilitation Barthel Index (Frühreha-Barthel-Index) at discharge. Supplementary moderator analysis revealed a differential effect of neglect severity on the cognitive outcome depending on the affected hemisphere.

**Conclusion:**

Data indicate a strong association between apraxia and visuospatial neglect and early rehabilitation outcomes after stroke.

## Introduction

Given the increasing prevalence of stroke, its debilitating effects, and the following socioeconomic burden, improving the effects of rehabilitation on functional and cognitive outcomes remains a challenge. To this end, previous studies intended to identify predictors of stroke outcome. Typical factors related to recovery after stroke are lesion size and location, age, sex, education, and depression [3, 45]. Furthermore, the stroke patients’ cognitive status may influence the functional outcome: While some studies found that general cognitive functioning impacted rehabilitation outcomes [26], others showed that the prevalence and recovery of cognitive deficits after stroke depended on the cognitive domain affected [10], which include orientation, speech, praxis, attention, visuospatial abilities, processing speed, and executive functions. Specifically, executive functions and problem-solving are predictive of motor outcome [17], while verbal memory and fluency predict future independence in ambulation, and visuo-constructive abilities predict community ambulation [37].

Among the neuropsychological stroke sequelae, visuospatial neglect is relatively well examined. Neglect is defined as a failure to report, respond, or orient to contralesional stimuli that is not caused by primary perceptual or sensorimotor deficits [25]. The reported prevalence of neglect is approximately 30% in acute left hemisphere (LH) stroke and 50% in acute right hemisphere (RH) stroke patients [7]. Notably, most studies on neglect exclusively investigated patients suffering from RH stroke. The majority of these studies documented relevant effects of neglect on the functional outcomes after stroke, e.g., slower recovery and prolonged rehabilitation [8], more significant caregiver burden [6], more severely impaired activities of daily living (ADL, [14]), and poorer functional outcome [29]. Importantly, most previous studies on neglect and stroke recovery focused on long-term effects [2].

Apraxia is the inability to perform specific and predefined actions or learned and purposeful movements. These impairments cannot be (fully) explained by sensory, motor, and other cognitive deficits, affecting task comprehension, stimulus recognition, or response implementation [9]. While most apraxia studies focused on patients with LH lesions, increasing evidence suggests that lesions to both hemispheres can result in apraxia [32]. The prevalence of apraxia is approx. 30–50% after LH and 8–20% after RH stroke [39]. Although some previous studies suggested an impact of apraxia on functional outcomes, the results remain ambiguous: According to some studies, apraxia led to more pronounced ADL impairments [11], a more significant caregiver burden [16], and a poorer functional outcome [46]. In contrast, other studies failed to show a relevant effect of bucco-facial or limb apraxia on functional outcomes after stroke [39]. It is noteworthy that the apraxia studies markedly varied about the sample size, the definition of apraxia, and the apraxia assessments. In this study, we operationalized apraxia by the affected motor domains (pantomime, imitation) and effectors (finger, arm/hand, bucco-facial) and assessed the apraxic deficits accordingly [13].

The objective of this study was to investigate the impact of apraxia and neglect on the cognitive and functional outcomes after LH and RH stroke [5] within the short period of the early rehabilitation programs (2 or 3 weeks, see below). Moreover, since most previous studies focused on one or only a few variables [35, 43] and stroke rehabilitation is a complex and multi-faceted process, we aimed to assess the effects of multiple variables on the cognitive and functional outcome after stroke. Therefore, relatively large left and right hemisphere stroke patient samples (75 LH stroke patients and 75 RH stroke patients) were tested, permitting the examination of potentially differential effects of neglect and apraxia on rehabilitation outcomes.

## Methods

### Setting and sample

This study followed the principles of the Declaration of Helsinki in its current version from October 2013 (Fortaleza, Brasilia). Data were analyzed retrospectively from the medical records compiled between November 2017 and October 2020 at the Department of Neurology of the University Hospital Cologne. All patients were enrolled in the Neurological-Neurosurgical Early Rehabilitation program for acute to early subacute stroke patients established in Germany [42]. Only patients with an Early Rehabilitation Barthel Index [41] of less than 30 are eligible for this program. The patients receive at least 300 min of therapy per day, including specialized nursing, physical, occupational, and speech therapy. On average, patients are included in the early rehabilitation program for 2–3 weeks before being transferred to standard in-patient or out-patient rehabilitation centers, their nursing homes, or their home. The usual duration of the early rehabilitation in our department is two weeks for patients without invasive interventions and three weeks for patients after invasive interventions (e.g., thrombectomy). There was some variation regarding the duration of the early rehabilitation program due to holidays, weekends, and extra days compensating for medically necessary pauses. In our sample of 150 acute to sub-acute stroke patients, 110 patients were enrolled in the 2-week program and 40 patients were enrolled into the 3-week program. Thus, most patients stayed in the program for 14 days only, where the behavioural and neuropsychological assessments were usually performed on day 2 and the last 2–3 days of the program.

**Fig. 1:**
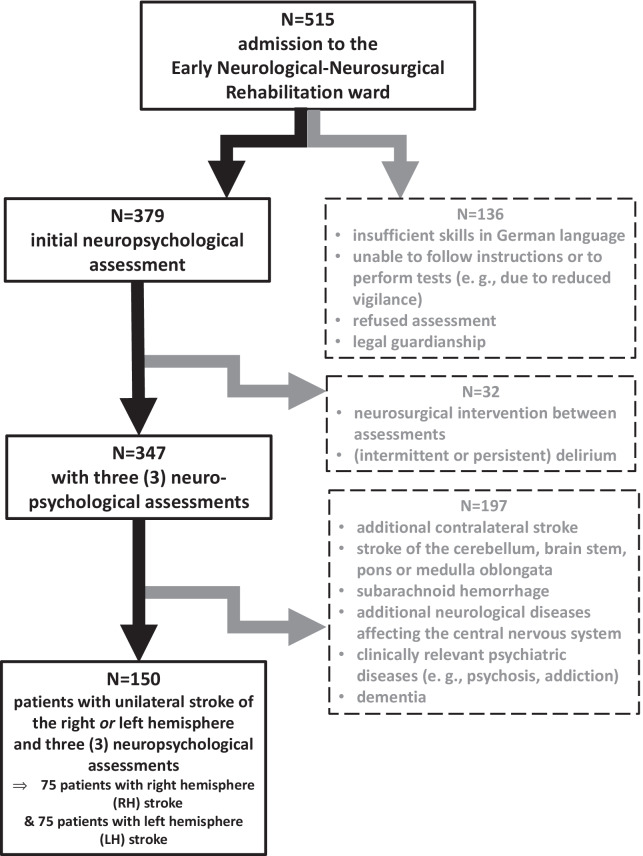
Composition process of the sample. Starting from 515 data sets, 150 remained in the final sample for evaluation (75 patients with left hemisphere (LH) stroke and 75 patients with right hemisphere (RH) stroke). Exclusion criteria are described on the right side in the dashed boxes

In total, the medical records of 515 patients were screened. An initial neuropsychological assessment was available for 379 patients since these patients had sufficient command of German, could follow instructions, did not refuse the assessment, and were not under legal guardianship (see Fig. 1).

A further 32 patients were excluded because a neurosurgical intervention or an (intermittent or persistent) delirium interfered with the neuropsychological follow-up assessments. Of the remaining 347 patients who underwent (at least in part) the three neuropsychological assessments at admission, at mid-term, and at discharge, 150 patients suffered from a hemispheric ischemic or hemorrhagic stroke and did not present with any of the following exclusion criteria: age > 90 years, additional contralateral stroke, a clinically relevant stroke of the cerebellum, brain stem, pons, or medulla oblongata, subarachnoid hemorrhage, other neurological diseases affecting the central nervous system, clinically relevant psychiatric diseases (e.g., psychosis, addiction, major depression), and dementia. Data of patients with previous strokes of the same hemisphere or an ipsilateral cerebellar stroke without motor symptoms (ataxia and intentional tremor) of the hand involved in the testing were not excluded, as well as patients suffering post-stroke depression. Table 1 shows the demographic and clinical features of the final sample (n = 150).

**Table 1:** Demographic and clinical information of the current stroke patients sample (n = 150)

Gender	Male	71 (47.3%)
	Female	79 (52.7%)
Age	Mean ± standard deviation	68.9 ± 13.9
	range	30–90
	Percentile 25	59
	Median	72
	Percentile 75	79
Education in years	Mean ± standard deviation	12.7 ± 3.5
	range	5–21
	Percentile 25	11
	Median	13
	Percentile 75	16
Affected hemisphere	Left	75 (50%)
	Right	75 (50%)
Stroke type	Ischemic	118 (78.7%)
	Hemorrhagic	32 (21.3%)
Stroke territory	Anterior cerebral arteria (ACA)	5 (3.3%)
	Middle cerebral arteria (MCA)	134 (89.3%)
	Posterior cerebral arteria (PCA)	1 (0.7%)
	ACA and MCA combined	6 (4%)
	MCA and PCA combined	4 (2.7%)
Time post-stroke (days) [at admission to the early rehabilitation program]	Mean ± standard deviation	5 ± 3.1
	Range	1–21
	Percentile 25	3
	Median	5
	Percentile 75	6

### Assessments

Handedness was assessed by the Edinburgh Handedness Questionnaire [38].

Three different parameters for functional outcome after stroke were employed: The National Institute of Health Stroke Scale, NIHSS), the German Early Rehabilitation Barthel Index (Frühreha-Barthel-Index, FRBI), and the composite score of the Functional Independence Measure (FIM) and Functional Assessment Measure (FAM). The NIHSS is a standard evaluator-based score to describe the severity of stroke symptoms [19]. It contains 11 items (each 0–max. 4 points); higher scores represent more severe impairments (total score 0–max. 42). The FRBI is based on the Barthel Index (BI) comprising 10 items (total score 0–max. 100, [34]) plus seven additional items with possible negative scores 0 to − 325 [41]. The FIM estimates disability in terms of caregivers’ burden considering motor and cognitive functions/deficits [20]. It covers 18 items (total score 0–max. 126). Commonly, the FIM is used together with the FAM [21], contributing additional information about psychosocial functions. The FAM consists of 13 items (total score 0–max. 42).

The cognitive status of the stroke patients was tested using the Cologne Neuropsychological Screening for Stroke Patients (KöpSS) [28], specifically with the KöpSS versions A and B at admission and discharge, respectively. The KöpSS can be performed even by severely impaired patients and examines the general cognitive performance level and seven relevant cognitive domains. Multiple subtasks assess each domain. Cut-off values exist for each domain and subtask. Overall, the cut-off value indicating cognitive impairment is set at ≤ 98 (of max. 108). In the current study, we applied a modified version of the KöpSS to avoid duplicate testing of cognitive functions by the KöpSS and the below-described apraxia and neglect assessments. Furthermore, KöpSS-items that required writing with the right hand or bimanual movements could not be performed by the right-handed stroke patients with motor impairments. The modified KöpSS (total score 0–max. 70) still encompassed 5 domains (orientation, language without the writing subtask, calculation, memory, attention and executive functions).

Apraxic deficits were assessed with the Cologne Apraxia Screening (KAS) [48] and the finger imitation test by Goldenberg [18]. The KAS (20 items, total score 0–max. 80, cut-off ≤ 76) comprises tasks that assess pantomiming the use of objects and imitation and include bucco-facial and arm/hand gestures resulting in four subtests (bucco-facial pantomime, arm/hand pantomime, bucco-facial imitation, arm/hand imitation). Patients are instructed to perform the pantomime corresponding to an object or imitate the presented gesture. All stimuli (objects, gestures to be imitated) are presented using photos. Patients with RH stroke were assessed with the KAS-R, a shorter version of the KAS (12 items, total score 0–max. 48, cut-off ≤ 46) with mirror-inverted stimuli that facilitate the spatial perception of the presented material [52]. For reasons of comparability, the raw scores of both KAS versions were transformed to a relative score for analysis (relative score = raw score * 100/maximally possible score). The Goldenberg Finger Imitation Test consists of 10 finger configurations that the patient should imitate with the ipsilesional hand in a mirror-like fashion after a demonstration by the examiner. For each item, 2 points are allocated for an immediate correct response, 1 point if the second attempt is successful, and 0 points if the patient fails on both attempts (total score 0–max. 20, cut-off-score ≤ 16).

**Fig. 2:**
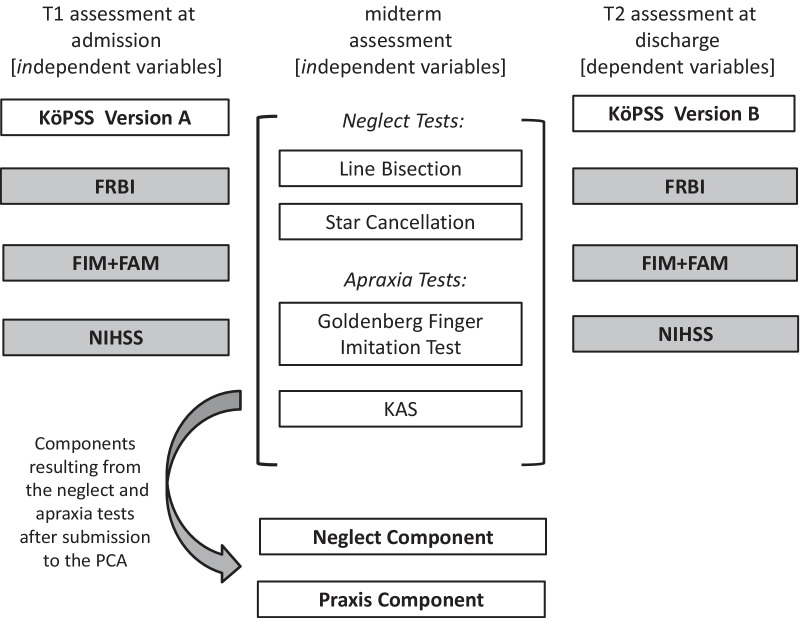
Conceptual model of the independent (T1 and midterm assessment) and dependent variables (T2). The diagram displays the different assessments performed after admission to the rehabilitation ward, at midterm and before discharge from the rehabilitation ward. Cognitive assessments are presented in white boxes and, functional assessments in grey boxes. The assessments at T1 and midterm served as independent variables and the assessments at T2 as the dependent variables in the statistical evaluation (here: multiple regression analysis with bootstrapped data)

The neglect assessment was based on two subtests of the Neglect-Test (NET) [15]—the German version of the Behavioral Inattention Assessment (BIT) [51]. The line bisection test (0–max. 9 points, cut-off ≤ 7) was used to assess a putative spatial perception bias, while the star cancellation test provided a score for visual exploration and allowed calculating a laterality quotient (LQ). Here, the absolute value of the LQ was considered to reflect spatial biases in either direction (LQ =|(hits contralesional-hits ipsilesional)/(hits contralesional + hits ipsilesional)|, range LQ: 0–1, cut-off ≥|0.2|; [14]). Figure 2 shows a conceptual schema of the dependent and independent variables.

### Design

Based on a screening of 515 hospitalized stroke patients from the University Hospital Cologne’s early rehabilitation ward, 150 stroke patients (75 left-hemispheric strokes, 75 right hemispheric strokes) fulfilled the inclusion criteria and were enrolled in this observational, longitudinal study. The cognitive status was documented at admission using the KöpSS Parallel-Version A and at discharge using the KöpSS Parallel-Version B. We also used the scores of the functional outcome scales at admission to and discharge from the Neurological-Neurosurgical Early Rehabilitation program (FRBI, NIHSS, and FIM/FAM). The assessments of apraxia (KAS, Goldenberg Imitation Test) and neglect (NET-line bisection, NET-star cancellation) took place at midterm.

### Statistical analysis

To allow for the maximal exploitation of the current data set by using more accurate methods we treated the data (e.g., FIM/FAM scores) like interval-scaled data in the current statistical evaluation. Furthermore, since we used the same method for all statistical evaluations, the current approach ensures comparability both within the study and with previous studies [8, 31, 36, 46].

To delineate the inherent structure of apraxia and neglect tests and reduce the number of independent variables, the two apraxia tests and the two neglect tests were evaluated using a principal component analysis (PCA). Two components reaching the Kaiser’s Criterion (eigenvalue > 1) were obtained and underwent a varimax rotation. Individual component scores were calculated by the regression method based on the two components.

Furthermore, we applied the wild bootstrap method, which is applicable if normality assumptions are not fulfilled (i.e., in case of exceeding skewness and heteroscedasticity). Moreover, based on 2000 samples, bias corrected and accelerated (BCa) confidence intervals were applied.

We applied four multiple regression analyses to identify relevant predictors of the cognitive and functional outcome scores at discharge. The dependent variables were the KöpSS score, the FRBI, NIHSS, and the FIM/FAM Composite Score at discharge (T2). Age, the Neglect and the Apraxia Component, and the score of the respective scales at admission (T1) were used as independent variables. All analyses were carried out using SPSS Version 27 (SPSS Inc., Chicago, Illinois).

Finally, to control potential influences of the affected hemisphere, four supplementary moderator analyses were performed using the PROCESS macro for SPSS (Version 3.5) [24]. The variables were used in analogy to the multiple regression analyses. In detail, the Neglect and the Apraxia Components were entered as independent variables, the remaining independent variables were classified as covariates, and the affected hemisphere (RH or LH) was added as a moderator.

Due to missing values, the number of cases entering the respective analyses differed and will be mentioned separately for each analysis. Note that the minimal number of cases was 106.

## Results

### Demographic and clinical characteristics of the patient sample

Out of the 139 patients who could be assessed with the Edinburgh Handedness Questionnaire, 133 (95.7%) were right-handed. The average time post-stroke at admission to the Neurological-Neurosurgical Early Rehabilitation program was 5.0 days (SD = 3.7). The interval between the assessments at admission and discharge was, on average, 12.2 days (SD = 3.9, range 5–23). The interval between the midterm and discharge assessments was 7.9 days (SD = 4.4, range 1–19). Visuospatial neglect was revealed in 45 (33.8%) of 133 patients by the line bisection test (mean = 7.14, SD = 2.82, range 0–9) and in 26 (19.1%) of 136 patients by the star cancellation test (mean = 0.16, SD = 0.33, range 0–1). Twenty-four (18.9%) of 127 patients who performed both the line bisection test and the star cancellation test showed impaired performance in one test and 20 patients (15.8%) in both neglect tests. Apraxia was diagnosed in 79 (54.1%) of 146 patients by the Goldenberg Finger imitation test (mean = 14.25, SD = 5.54, range 0–20) and for 100 (70.9%) of 141 patients by the KAS (mean = 81.12, SD = 21.6, range 0–100). Forty-two patients of 136 patients (30.9%) who performed both the Goldenberg Finger imitation test and the KAS scored below the cut-off in one of the apraxia tests, 63 (46.3%) patients were impaired in both apraxia tests.

Following previous studies [30, 50], we diagnosed apraxia and neglect when patients scored below the cut-off in at least one test. Accordingly, 105 (of 134 patients, 77.2%) patients were apraxic, and 44 (of 127 patients, 34.6%) suffered from neglect.

A significant improvement was observed for the cognitive and all functional scales during the rehabilitation period (Table 2).

**Table 2:** Improvement in the cognitive and functional outcome scales during the early rehabilitation and distribution characteristics

	KöpSS n = 120	FRBI n = 149	FIM + FAM n = 137	NIHSS n = 146
*Score at admission (T1)*				
Mean	49.42	0.77	66.07	14.15
SD	13.05	28.60	24.96	4.51
Median	45	10	59	14
Range	17.5–66.5	− 75 to 30	30–162	3–31
Percentile 25	35.5	0	49	6
Percentile 50	45	10	59	10
Percentile 75	55	15	75,5	13
*Score at discharge (T2)*				
Mean	66.07	25.00	89.65	9.73
SD	13.79	28.39	35.52	4.30
Median	51.75	25	84	10
Range	13.5–70	− 75 to 100	45–210	0–22
Percentile 25	40.75	15	65	6
Percentile 50	51.75	25	84	10
Percentile 75	60	40	104	13
*Comparison (T1 and T2) of mean values*				
t	**7.487**	**11.062**	**14.482**	**− 16.067**
df	119	148	136	145
p	** < 0.001**	** < 0.001**	** < 0.001**	** < 0.001**

Mean values, standard deviations, median values, range and quartile scores of the cognitive and functional outcome scores in the assessments at admission (T1) and discharge (T2). The improvements between T1 and T2 were examined with t-tests for dependent samples. Significant results are presented in bold. The significance of all four comparisons were confirmed by nonparametric testing

Entering the scores of the two apraxia tests and the two neglect tests into a Principal Component Analysis (PCA) revealed two components that fulfilled the Kaiser criterion (eigenvalue > 1), explaining 79.8% of the variance (Table 3). A subsequent varimax rotation revealed that the first component (hereafter: Neglect Component) primarily represented the scores of the neglect tests, while the second component reflected the apraxia test scores (hereafter: Apraxia Component). Individual component scores for further calculations were obtained using the regression method. Higher scores indicate better performance (i.e., less severe apraxia or neglect).

**Table 3:** Results of the principal component analysis (PCA) of the neglect and apraxia assessments

	Neglect component	Apraxia component
Line bisection	**0.829**	0.339
Star cancellation	**− 0.932**	0.05
Goldenberg finger imitation	0.343	**0.759**
KAS (percentage)	− 0.009	**0.904**
*Eigenvalue*	*2.137*	*1.053*
*Explained variance*	*53.4%*	*26.3%*

The component matrix shows the loading patterns of the neglect and apraxia test scores on the two components extracted from PCA after varimax rotation. Relevant loading scores are highlighted in bold. Eigenvalues and explained variance are listed below

### Multiple regression analysis

For all scales at discharge, the multiple regression analysis yielded a strong predictive value of the respective scale at admission. The KöpSS score at admission predicted the KöpSS score at discharge (b = 0.425, t = 6.714, p < 0.001, n = 100, Table 4). The initial assessment of FIM/FAM predicted the final variance of the FIM/FAM compound score (b = 1.187, 16.324, p < 0.001, n = 113). Likewise, the initial NIHSS score predicted the final NIHSS score (b = 0.699, t = 9.075, p < 0.001, n = 106). Finally, the initial FRBI score predicted the final FRBI measure (b = 0.55, t = 5.858, p < 0.001, n = 121).

**Table 4:** Explained variance and regression model characteristics of the multiple regression results

Predictors					
	Score at T1	Apraxia component	Neglect component	Age	Education
*KöpSS (n = 100)*					
Regression model	b = 0.425 t = 6.714	b = 5.084 t = 5.378	–	b = − 0.166 t = − 3.398	b = 0.373 t = 2.011
Explained variance **68.2%**	**p < 0.001** [0.307,0.555]	**p < 0.001** [2.954,6.99]		**p < 0.01** [− 2.65,− 0.075]	**p < 0.05** [0.057,0.68]
*FRBI (n = 121)*					
Regression model	b = 0.55 t = 5.858	–	b = 5.668 t = 2.34	–	–
Explained variance **34,5%**	**p < 0.001** [0.256,0.859]		**p < 0.05** [0.719,11.334]		
*FIM/FAM (n = 113)*					
Regression model	b = 1.187 t = 16.324	–	–	–	–
Explained variance **73,8%**	**p < 0.001** [1.014,1.484]				
*NIHSS (n = 106)*					
Regression model	b = 0.699 t = 9.075	–	–	–	–
Explained variance **51,6%**	**p < 0.001** [0.548,0.853]				

The table displays variance explained by the multiple regression analysis and the regression model characteristics (unstandardized regression coefficient, t-statistic, level of significance, and bias-corrected and accelerated (BCa) confidence intervals 95% CI)

The table shows only significant results

**Fig. 3:**
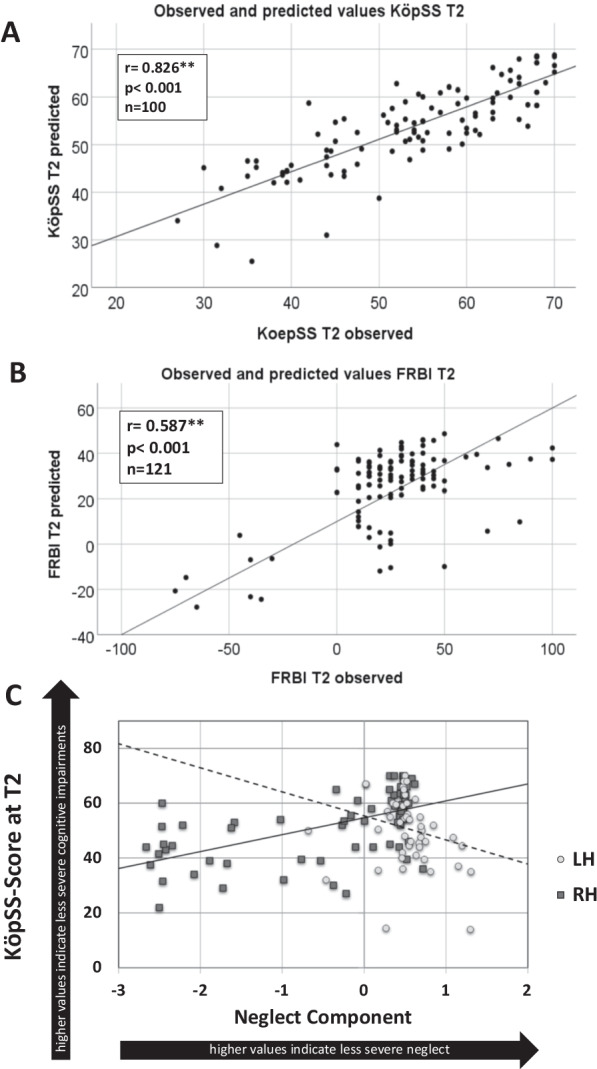
Distributions of observed and predicted scores for the cognitive and functional outcomes and the moderation effect of the hemispheres for cognitive outcome. **A**, **B** Visual depiction of the values predicted by the multiple regression models and actually observed values for cognitive (KöpSS at T2, **A**) and functional (FRBI at T2, **B**) outcome. For both outcomes, there was a significant correlation between predicted and observed values. **C** Cognitive outcome (KöpSS) at discharge (T2) predicted by the Neglect Component for LH (n = 41) and RH stroke patients (n = 59). The X-axis displays individual component scores of the Neglect Component as z-transformed scores. The y-axis shows the KöpSS scores at discharge (T2). A significant moderator effect revealed an impact of the Neglect Component for RH stroke patients only (depicted by the continuous trend line)

Besides the dominant effects of the initial behavioral scores, we found significant effects on cognitive and rehabilitation outcomes for the components reflecting apraxia and neglect severity. In particular, the Apraxia Component accounted for the KöpSS score variability (b = 5.084, t = 5.378, p < 0.001; see Fig. 3A). In contrast, the Neglect Component showed an effect (b = 5.668, t = 2.34, p < 0.05; see Fig. 3B) on the discharge FRBI, with the latter effect being independent of the affected hemisphere. A significant negative impact of age (b = −0.166, t = −3.398, p < 0.01) and education (b = 0.373, t = 2.011, p < 0.05) was found only for the KöpSS score at discharge.

### Moderator analysis of putative effects of the hemisphere affected by the stroke

Supplementary moderator analysis revealed a significant differential effect of the affected hemisphere on the Neglect Component when predicting the KöpSS score at discharge. As illustrated in Fig. 3C, more severe neglect (as indicated by a lower Neglect Component score) predicted 2.9% of the variance of the final KöpSS score (F(1,93) = 9.45, p < 0.01, n = 100) for the patients suffering from a RH but not LH stroke. No difference between the affected hemispheres was found for the Apraxia Component (or any other scale/ score).

## Discussion

Beyond the expected effects of the baseline (T1) values on a given scale at admission, cognitive deficits of praxis (apraxia) and attention (neglect) affected the cognitive and functional outcomes at discharge. Apraxia severity predicted the final KöpSS. Neglect had a significant effect on the FRBI. Moreover, higher age and lower education negatively affected the cognitive outcome in the early rehabilitation after stroke. The current study replicates previous results of multifactorial models, where neglect explained functional outcome independently or in addition to other variables [27]. The only study that revealed no effect of neglect on (functional) outcome after stroke [40] was based on a neglect assessment with a single item. Thus, cognitive deficits after stroke should be assessed with multiple tests, preferably covering different cognitive domains, as in the current study.

The amount of variance explained by the Neglect Component (3.7%) was smaller than in other studies [31].

Eight items of the Barthel Index, a part of the FRBI, require spatial navigation of directional movements. Since spatial navigation is hampered by neglect, neglect was shown to have an unfavorable effect on transfer and locomotion [4, 36].

We selected the star cancellation and line bisection tests for neglect assessment since previous studies revealed that these neglect tests predicted long-term functional independence after stroke [1, 33], with more pronounced effects for the cancellation tests [31]. Note that both tests mainly assess peripersonal neglect, the space within reach [22]. In previous work [2], peripersonal neglect correlated more with long-term functional outcomes after stroke than personal or extrapersonal neglect.

Notably, the factor “affected” hemisphere moderated the effect of neglect severity on cognitive outcome in that a significant negative impact of the neglect component could be found only after RH stroke.

In contrast, apraxic deficits had no impact on functional outcome at discharge of the Neurological-Neurosurgical Early Rehabilitation program. The result is consistent with previous studies conducted in the acute phase after stroke [39]. However, evidence for predictive effects of apraxia severity on functional outcome and simple activities of daily living can be found in previous studies of patients in sub-acute or chronic phases after stroke [12, 23]. In our sample, consisting of patients in an acute to sub-acute phase after stroke, a predictive role of apraxia severity was confirmed for cognitive, but not for functional outcome.

The observed negative effect of age on cognitive stroke recovery has repeatedly been mentioned in the literature. Recently, these effects were also observed for acute stroke patients [45]. A positive impact of education on stroke outcome is known and widely discussed as an indicator of “cognitive reserve” [44], a term comprising acquired mental capacities that have a moderating, protective influence in case of brain damage due to a stroke.

### Limitations

Apraxia and neglect are multi-faceted syndromes. Because of limited statistical power, this study could not account for potential differences between the different facets of apraxia, e.g., bucco-facial versus limb apraxia [32], or neglect, e.g., peripersonal versus extra-personal neglect [49]. The current statistical power also precluded an analysis of subscales of the cognitive and functional outcome scores. Finally, further studies are warranted to investigate other common cognitive sequelae of LH stroke like aphasia [50] or RH stroke like anosognosia [47]. A further limitation of our study is that data about the long-term outcome of the current stroke patient sample are not available. Future studies are warranted that investigate the important relationship between improvements in the early rehabilitation after stroke (and their predictors) and the functional and cognitive long-term outcome after stroke. Concerning the statistical methods applied, we know that the FIM/FAM is an instrument with an ordinal Likert scale like many clinical questionnaires. However, to maximally exploit the current data set and to ensure the comparability with previous studies, we treated the FIM/FAM data like interval-scaled data in the current statistical evaluation.

## Conclusions

This study’s results underline the importance of assessing apraxia and visuospatial neglect in the early subacute post-stroke phase. The current findings also emphasize the need for developing appropriate therapeutic approaches for the cognitive sequelae of a hemispheric stroke to ameliorate the harmful effects of apraxia and neglect on early stroke rehabilitation.

## Data Availability

Data are available from the authors on reasonable request.
